# Valorization of Tomato Waste as a Source of Carotenoids

**DOI:** 10.3390/molecules26165062

**Published:** 2021-08-20

**Authors:** Sonia Trombino, Roberta Cassano, Debora Procopio, Maria Luisa Di Gioia, Eugenio Barone

**Affiliations:** 1 Department of Pharmacy and Health and Nutrition Sciences, Department of Excellence L. 232/2016, Edificio Polifunzionale, Università della Calabria, 87036 Rende, Italy; Sonia.trombino@unical.it (S.T.); Roberta.cassano@unical.it (R.C.); debora.procopio@unical.it (D.P.); 2 Department of Biochemical Sciences “A. Rossi-Fanelli”, Sapienza University of Rome, Piazzale Aldo Moro, 00185 Rome, Italy

**Keywords:** food waste valorization, carotenoids, value-added products, recycling, tomato byproducts, lycopene, antioxidants

## Abstract

Fast-accumulating scientific evidence from many studies has revealed that fruits and vegetables are the main source of bioactive compounds; in most cases, wastes and byproducts generated by the food processing industry present similar or a higher content of antioxidant compounds. In recent years, the ever-growing amount of agricultural and food wastes has raised serious concerns from an environmental point of view. Therefore, there is an increasing interest in finding new ways for their processing toward safely upgrading these wastes for recovering high-value-added products with a sustainable approach. Among food waste, the abundance of bioactive compounds in byproducts derived from tomato suggests possibility of utilizing them as a low-cost source of antioxidants as functional ingredients. This contribution gives an overview of latest studies on the extraction methods of carotenoids from tomato waste, along with an evaluation of their antioxidant activity, as well as their industrial applications.

## 1. Introduction

Biowaste products, which remain at the end of a certain production process, are a great resource which, if not reused, can be a cost for the industry that produces it, as well as an environmental problem. It serves as a reminder of the waste from the vegetable industry or agriculture in general ca, as well as their associated costs. In the past, people added these byproducts as compost to the soil for agricultural purposes, thus allowing the recycling of nutrients. Today, instead, because of the huge increase in the accumulation of large amounts of waste matter, reducing waste is among the efforts to relieve the pressure on natural resources and move toward more sustainable food systems. However, the “waste” problem assumed as environmental sustainability is not a topic of recent attention; Directive 2008/98/EC proposed strengthening the byproduct market and, in this way, reducing the use of raw materials and the production of waste.

Key challenges relate to moving beyond the perception of ‘waste as a problem’ to ‘waste as a resource’ through the search for new uses in different fields such as cosmetics, pharmaceuticals, bioenergy, and recovery of ingredients useful in enrichment and preservation of food [1].

Tomato, *Lycopersicon esculentum*, is the second most important vegetable crop worldwide and is a key component of the Mediterranean diet; its consumption is directly associated with reducing the risk of inflammatory processes, different chronic diseases, and carcinogenesis, as well as the inhibition of the oxidation of low-density lipoproteins, thus helping to lower the level of blood cholesterol [2].

Over 130 million tons are processed every year, and approximately eight million tons represent the waste generated as estimated by the World Processing Tomato Council (WPTC) [3]. In fact, a considerable number of produced tomatoes are not suitable for fresh consumption due to unacceptable color, maturity, or shape, thus representing an economic loss for producers and a negative environmental impact [4]. Additionally, large quantities of tomato peel residues are generated from the processing industry. Therefore, the recycling of tomato waste is currently among the top environmental stakes, and alternative uses need to be proposed.

The solid residue remaining after the industrial processing of tomatoes, i.e., tomato pomace, consists of large amounts of tomato peels and seeds that currently find use as animal feed and fertilizers or are sent to landfill [5,6]. However, it is still rich in highly beneficial phytochemicals such as carotenoids, phenolic compounds, and vitamins, among which lycopene is the most important antioxidant present in the ripened tomato, representing 80–90% of the total pigments [7,8]. Many epidemiological data support a correlation between carotenoids, especially lycopene intake, and several nutritional and health benefits, including the prevention of carcinogenesis and cardiovascular diseases [9,10,11]. In addition, phenolics are recognized for their antimicrobial and antioxidant activity and for their contribution in preventing various oxidative stress-associated diseases [12,13].

In this line, given their antioxidant or nutritional properties, numerous approaches have been proposed for the valorization of the unused parts of tomato in various sectors, including the recovery and isolation of carotenoid compounds to be used for the formulation of functional foods, as well as for pharmaceutical and cosmetic products, instead of chemically synthesized molecules [14,15,16,17]. On the other hand, the conventional extraction procedures to obtain bioactive compounds suffer from a huge amount of toxic organic solvents, low extraction selectivity, and decomposition of thermolabile compounds. Therefore, to overcome these limitations, the development of innovative and sustainable approaches in the extraction of these substances must be applied.

**Figure 1:**
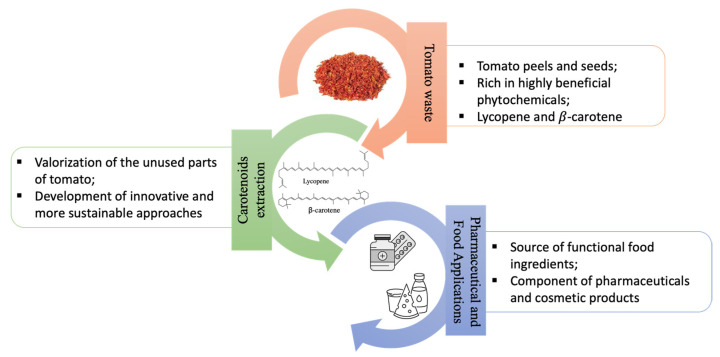
Main scope of the review: carotenoid extraction procedures from tomato waste and industrial applications.

In light of the abovementioned arguments, the present review addresses the most recent developments in the extraction of bioactive antioxidant compounds from tomato processing waste, evaluating the possible application on an industrial scale. A focus on its valorization in the food and nutraceutical industry is also presented (Figure 1).

## 2. Alternative Processes for Tomato Carotenoid Extraction

**Figure 2:**
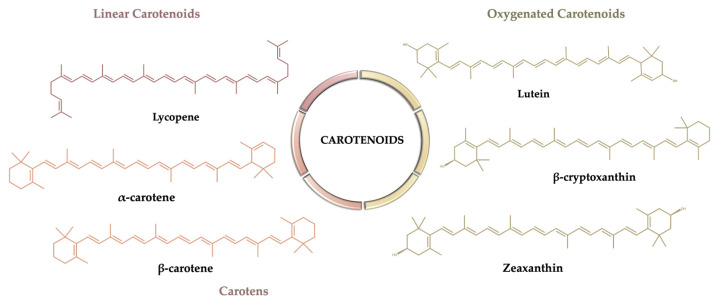
Chemical structures of the main carotenoids that occur in tomatoes.

Carotenoids are natural lipid-soluble pigments which are accumulated in the chloroplasts and chromoplasts in the outer skin layer during the ripening process of tomatoes [18]. Considering their structure, they are divided into two major groups: carotenes, which are hydrocarbon carotenoids that are either cyclized (such as α-carotene and β-carotene) or linear (lycopene), and oxygenated carotenoids called xanthophylls (such as lutein, zeaxanthin, and β-cryptoxanthin) (Figure 2).

When present in the human diet, these bioactive compounds act as cardiovascular disease preventers, anticarcinogens, and immune system regulators [19]. They have a significant role in human health by serving as important dietary sources of vitamin A and acting as biological antioxidants, trapping reactive oxygen species (ROS) and reducing oxidative damage to lipids, proteins, and deoxyribonucleic acid (DNA) [20]. Moreover, they interact synergistically with other antioxidants to protect cells and tissues from oxidative damage [7].

Indeed, the fundamental carotenoids that occur in tomatoes are lycopene and β-carotene. In peel byproducts, lycopene content corresponds up to 90% of the total carotenoids; instead, β-carotene represents the major carotenoid in seeds. Furthermore, most of the literature data agree on the fact that the highest quantities of carotenoids are found in tomato waste. In particular, tomato peel typically contains the highest lycopene content (about 377 μg/g), followed by industrial waste (about 175 μg/g) and whole tomato (about 82 μg/g), on a dry weight basis [21].

The lycopene content in tomatoes depends on the variety, geographic location, cultivation technique, and climatic conditions, increasing as the fruit ripens [22,23,24]. Lycopene, thanks to its particular acyclic tetraterpenic structure with 13 double bonds, 11 of which are conjugated (Figure 2), is considered one of the most powerful antioxidants; in fact, according to in vitro studies, its ability to quench singlet oxygen is twice as high as that of β-carotene. The extended conjugated polyene system is the key to the biological activity of lycopene, which includes its susceptibility to oxidative degradation [25].

It is responsible for protecting cells from oxidative damage, thereby diminishing the possibilities of chronic diseases; for this reason, it is present as an important component in pharmaceutical products for the treatment of prostate and digestive tract cancers [26].

Furthermore, the contemporary presence of β-carotene, also known as provitamin A, synergistically increases the antioxidant effect in the tomato [27]. In addition, lycopene, along with β-carotene, which is an FDA-approved supplement with GRAS status (generally regarded as safe), is also an authorized natural pigment for numerous food products [28,29].

Due to their association with human health, the demand for high-purity, well-characterized, and low-cost natural carotenoids is constantly increasing, and their market is expected to grow from 1.5 billion USD in 2019 to 2.0 billion USD by 2026, recording a compound annual growth rate of 4.2% during the estimate period [30]. However, their synthesis is not economically advantageous, and, above all, the Food and Drug Administration has only approved lycopene extracted from tomatoes as an additive and not that synthesized or extracted from other natural sources [31]. Therefore, the extraction from tomato industrial byproducts constitutes a very valid alternative. This may lead to a revalorization of the isolated substances in line with the increasing trend toward utilization of food processing byproducts as a source of functional food ingredients or as component of pharmaceuticals and cosmetic products [32].

Established protocols for lycopene extraction from tomato utilize traditional solvent extraction methods based on the use of conventional organic solvents and different combinations of solvent mixtures [33,34,35]. A recent study showed that highly efficient solvents for the direct recovery of lycopene from tomato peels can be easily prepared using a mixture design approach [36]. A mixed-polarity solvent mixture composed of *n*-hexane–ethanol–acetone resulted in a lycopene extraction yield higher than 95%. The solvent choice was made on the basis of the high-affinity characteristic for lycopene, in the case of hexane, whereas ethanol and acetone were selected because of their ability to produce swelling of cellulosic materials, thus enhancing solvent penetration. This mixture allowed the production of a tomato oleoresin with high lycopene content (about 13% *w*/*w*) and antioxidant capacity (1582 μmol TE/g).

**Figure 3:**
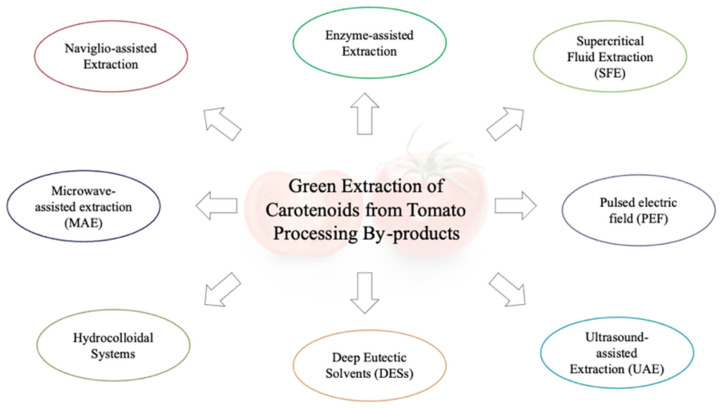
Green extraction methods of tomato carotenoids.

Discarded tomato skins from the production of tomato juice have been found to be the best source for lycopene extraction. It must be considered, however, that some factors such as drying conditions for the tomato pomace, light exposure, and tomato cultivar could affect carotenoid recovery. Furthermore, carotenoid degradation can be a serious obstacle when developing an extraction method. In fact, the conjugated double bonds in the carotenoid skeleton structure make them susceptible to oxidation in the presence of heat, light, unsaturated fats, peroxides, and some metals [37]. Harsh reaction conditions can cause carotenoid isomerization from the natural *trans* state to the *cis* state, resulting in reduced color intensity and vitamin A activity [38]. To these drawbacks must be added the fact that existing extraction procedures are time-consuming and require the use of large quantities of common organic solvents that are recognized to be a personnel and environmental issue. A reduction in their use is one of the main themes in green chemistry. Thus, more and more eco-compatible extraction processes operating under mild conditions and allowing the solvent to overcome mass-transfer limitations in the plant matrix have been investigated [39] (Figure 3).

### 2.1. Enzyme-Assisted Extraction

Recent studies have demonstrated that enzyme-assisted extraction can cause the hydrolysis of the polysaccharide network of the primary cell wall of tomato where the pigments accumulate, favoring solvent penetration and leading to a noteworthy improvement in the release of carotenoids, as well as a reduction in extraction time and temperature [40,41,42]. Enzyme-assisted extraction is not so much considered a true extraction technique, but instead an effective pretreatment. Prokopov et al. [43] suggested that the pretreatment of tomato peels with mixed cellulolytic and hemicellulolytic enzymes is a good approach for carotenoid recovery. In particular, they showed that the pretreatment of Bulgarian tomato peels with a mixture of cellulase (100 U·g^−1^) and endo-xylanase (400 U·g^−1^) for 4 h at 50 °C resulted in an up to 1.6-fold increase in the carotenoid extraction yield. Accordingly, Catalkaya and Kahveci [44] attempted the optimization of lycopene recovery from industrial tomato waste by selecting the most suitable solvent and enzyme. The effect of different solvents (such as acetone, ethyl acetate, ethanol, and their 1:1 combination) on the total phenolic content and antioxidant capacity of the extracts was evaluated. The results revealed that the polarity of the solvent plays a crucial role in the extraction process; in fact, the combination of acetone and ethyl acetate with cellulolytic and pectinolytic enzymes afforded an oleoresin with improved antioxidant properties, as well as the highest lycopene recovery (9.16 ± 3.00 mg/g oleoresin) and one of the highest red color intensities.

However, enzymatic methods are not yet totally exempt from hazardous solvents use and still have limited recovery yields of carotenoids [45].

### 2.2. Supercritical Fluid Extraction

Among the environmentally safe technologies, special attention is paid to supercritical fluid extraction (SFE) since the use of a fluid at a pressure and temperature near or above its critical value, while enhancing the solvating power of the solvent, offers the advantage of a clean technique. Several compounds can be used as supercritical fluids, but carbon dioxide (CO_2_), being nontoxic, nonflammable, economic, and easily available, has been successfully used as the solvent for the extraction (SC-CO_2_). It is also important to emphasize that it is labeled as “generally regarded as safe” (GRAS) by both the Food and Drug Administration (FDA) and the European Food Safety Authority (EFSA) [46].

The main objective of the reported SC-CO_2_ methods is the optimization of parameters such as temperature, pressure, CO_2_ flow rate, extraction time, sample moisture content, and sample particle size [47,48]. Kehili and coworkers [49] used the SC-CO_2_ technique to extract lycopene and β-carotene as oleoresin from Tunisian industrial tomato peels. The relative extraction yields varied from 32.02% to 60.85% for lycopene and from 28.38% to 58.8% for β-carotene, and only the extraction temperature had a substantial effect on the process. They also investigated the influence of the supercritical CO_2_ extraction conditions on the extract antioxidant capacity using the quenching activity of the free chromogenic radical, 2,2-diphenyl-1-picrylhydrazyl (DPPH). Interestingly, the extracted oleoresin exhibited competitive antiradical activity with the synthetic antioxidant, butylated hydroxytoluene (BHT). Results showed that the industrial tomato peel byproduct is a potential source of highly antioxidative, solvent-free, and lycopene- and β-carotene-enriched oleoresin with promising applications in food and pharmaceutical industries. Furthermore, SC-CO_2_ was compared to the conventional extraction of lycopene by maceration in terms of energy used, and results showed that energy consumption was lower for SC-CO_2_.

Yet, despite the high target specificity and the green features of the procedure, the high pressure required to keep the fluid in a supercritical state is considered a limiting factor, which makes the whole process not economically viable on a large scale. Moreover, the final extract contains only a limited percentage of lycopene (max 14% (*w*/*w*)) because of the simultaneous extraction of lipids.

Recent studies have found that the addition of a cosolvent can improve the solubilization of the carotenoids, thus increasing the SC-CO_2_ extraction [50]. De Andrade Lima et al. [51] evaluated the possibility of using the SFE methodology with ethanol as a cosolvent. The preference for this particular entrainer was due to its low price and toxicity, as well as its ability to increase the polarity of CO_2_, compared to other polar solvents, such as methanol or acetone. Total carotenoid recovery was greater than 90% *w*/*w*, and lycopene was recovered efficiently (~95%) due to the high moisture content of tomato peels. Furthermore, tomato peel extracts showed a high antioxidant activity (88%) due in particular to the use of ethanol which, increasing the polarity of CO_2_, led to the simultaneous extraction of carotenoids and phenolic antioxidant compounds [52].

### 2.3. Pulsed Electric Field Extraction

In order to improve the extractability of carotenoids from tomato peels, Pataro et al. [53] reported the use of a pulsed electric field (PEF) for the pretreatment of whole tomato fruits. The researchers investigated the effects of different electric field strengths and steam blanching (SB) temperatures on the cell disintegration index of peel tissue, as well as on the total content and composition of carotenoids and antioxidant activity of the extracts. The results of this study suggest that the cell disintegration induced at the cuticular level by the electrical and/or thermal treatment improves the penetration of the solvent into the cytoplasm and the subsequent mass transfer of the solubilized intracellular pigments, thus intensifying the extractability of carotenoids. Application of a PEF treatment intensity (>0.75 kV/cm) in combination with steam blanching of tomato fruits at 60 °C might further enhance the cell disintegration level of peel tissues with a synergistic effect on promoting the extraction yield of intracellular carotenoids. The combined methodologies afforded a very high yield in total carotenoids (up to 188% for PEF and 189% for SB) and antioxidant power (up to 372% for PEF and 305% for SB) with respect to the peels from untreated tomatoes. In addition, HPLC analyses revealed not only that lycopene was the predominant carotenoid in peel extracts, but also that the application of electrical and/or thermal pretreatment does not result in any isomerization or degradation of lycopene.

PEF technology enables degradation, as well as reduces the time, energy cost, and solvent consumption [54,55]. Nevertheless, further studies are required to fully validate the implementation of PEF technology at an industrial scale.

The same research group later investigated [56] the influence of pulsed electric field (PEF) pretreatment on the recovery yield of lycopene in either acetone or ethyl lactate from industrial tomato peels residue. They demonstrated that the application of PEF treatment (5 kV/cm, 5 kJ/kg) prior to solid–liquid extraction significantly enhanced the extraction rate (27–37%), the lycopene yields (12–18%), and the antioxidant power (18.0–18.2%) in both environmentally friendly solvents. However, acetone gave the highest lycopene yield, suggesting a better ability of this solvent to penetrate the plant cells of wet tomato peel tissue and to solubilize intracellular lipophilic compounds.

### 2.4. Ultrasound-Assisted Extraction

The increasing demand for clean and green extraction techniques has also led to the application of ultrasounds [57]. The ultrasound waves by means of a phenomenon called acoustic cavitation cause a violent collapse of gas bubbles present in the solvent, simplifying the release of the target compounds from the matrix to the solvent, while also lowering the environmental impact. Furthermore, being a nonthermal technology, ultrasound has exhibited particular efficacy in increasing the extraction of heat-labile compounds [58]. Silva et al. [59] carried out an ultrasound-assisted extraction (UAE) of lycopene from the tomato pomace using, for the first time, an ecofriendly solvent mixture containing ethyl lactate and ethyl acetate [60,61]. The optimized extraction conditions (63.4 °C, 30% (*v*/*v*) EA in solvent mixture, 100 mL/g solvent: sample ratio, and 20 min), achieved a high lycopene yield of 1334.8 ± 83.9 μg/g in a relatively short time with a mild temperature. The yield without ultrasound at optimum conditions was 1209.5 μg/g which was about 9% lower than with UAE, showing that the applied ultrasound treatment promoted an increase in extraction yield and indicating the effectiveness of the green solvent mixture proposed for lycopene extraction. Said and collaborators [62] further demonstrated that applying the combined treatment of freeze-drying and ultrasonication (45 min at 50 Hz) increased the yield of extracted lycopene from industrial tomato waste 4.12-fold.

The interest of Silva et al. toward the development of more and more ecofriendly methodologies led the research group to evaluate the effectiveness of deep eutectic solvents (DESs) in the ultrasound-assisted extraction of lycopene [63]. DESs represent a new generation of solvents that are emerging as the green solvents of the 21st century, mostly based on low-transition-temperature mixtures of cheap and easily available components. The possible combination of the starting materials to prepare the eutectic solvents is virtually countless; therefore, they are enjoying great success and interest as solvents for extracting bioactive compounds [64,65,66].

In this study, the extraction process was performed under ultrasound assistance using hydrophobic deep eutectic solvents that were prepared using dl-menthol as a hydrogen-bond acceptor (HBA) and lactic acid as a hydrogen-bond donor (HBD). The optimized lycopene extraction yield achieved was 1446.6 mg/kg dry weight, which was higher than an optimized extraction from the same tomato pomace using a solvent mixture of ethyl acetate and ethyl lactate (1334.8 mg/kg dry weight) [58]. This extraction procedure is very appealing; the used DESs are nontoxic and biodegradable, thus not affecting the quality or safety of the extract, the purification step can be eliminated, and the lycopene-rich extract can be directly applied in food or products, encouraging its industrial application [67].

### 2.5. Other Extraction Methods

Another promising green approach was recently reported by Nagarajan et al. [68]. The procedure relies on the formation of a hydrocolloidal system by simple water-induced complexation of lycopene in the presence of pectin, a bioactive compound also present in tomato pomace. The colloidal complexes were then recovered by sedimentation or centrifugation. The complexation method was accomplished in a single step with a minimum amount of organic solvent and short extraction time. The maximum recovery was 9.43 mg of carotenoid fractions/100 g of tomato pomace, while the purity of the carotenoid-rich fractions was 92%. The antioxidant level of carotenoid extracted by complexation were 47.7% higher that by conventional solvent extraction, as determined using the DPPH assay. It was noticed that the pectin in the recovered complex played a significant role in protecting the carotenoid pigments entrapped in the complex, thus preserving the antioxidant capacity of the compound. The applicability of this extraction method can be extended to other fruit-processing wastes that are naturally abundant in both pectin and carotenoids.

Furthermore, microwave-assisted extraction (MAE) has emerged in recent years as an exceptional energy resource to promote ecofriendly extractions while greatly shortening the extraction times, reducing solvent usage, and increasing the yield and quality of product [69]. In this sense, this technique may provide a solution to the use of high temperature or long heat treatment, which can cause the degradation of lycopene. Lasunon et al. very recently investigated the effect of MAE on bioactive compounds from industrial tomato waste and the antioxidant activity of the obtained extract [70]. Generally, the efficiency of microwave extraction is affected by the identification of the optimal extraction parameters; therefore, different conditions including microwave powers of 180, 300, and 450 W and extraction times of 30, 60, and 90 s were evaluated using 95% ethanol with a temperature not exceeding 77 °C. According to the authors, the microwave power of 300 W for 60 s provided the highest *trans*-lycopene and β-carotene recovery (5.74 mg lycopene/100 g and 4.83 mg β-carotene/100 g). This result was probably due to the higher microwave power, which causes a decrease in the interaction between the target compound and the sample matrix, leading to an improvement in the extractability of the target compounds. Nevertheless, an increase in microwave power levels (450 W) caused a degradation of carotenoids during the extraction. Instead, the highest DPPH radical scavenging was observed when a microwave power of 180 W for 90 s was applied.

Alongside these green techniques, the use of the Naviglio extractor is gaining interest as an alternative process [71]. The technology works on a new extractive principle that is based on the fact that, in a suitable solvent, generating a gradient pressure between the inner and the outlet of solid matrix, a forced extraction can be produced of the not chemically bound compounds contained in the solid matrix [72]. Starting from tomato skin waste, the extractive process enabled the transfer of lycopene in the form of molecular aggregates in deionized water avoiding thermal stress. The lycopene recovery percentage was lower than that obtained using the traditional extraction method; nevertheless, the chromatographic analysis by high-performance liquid chromatography of the lycopene extract obtained showed a purity of more than 98% (*w*/*w*). Therefore, considering that, at the end of the process, water can be recovered by filtration and reused later, and that exhausted tomato-waste can be easily dried at room temperature and further used in agriculture or as animal feed, the process becomes attractive and scalable for industrial application. The latest and most innovative works are summarized in Table 1.

## 3. Protection of Tomato Carotenoids Prior to Industrial Application

A successful extraction technology for carotenoid recovery from tomato pomace can significantly improve the economic aspects of the tomato industry in addition to obtaining lycopene, one of the most potent antioxidants for formulating health supplements and improving the shelf life of numerous food products [5]. On the other hand, thanks to the characteristic range of colors from red to orange and yellow, tomato carotenoids are increasingly being applied as natural colorants [73]. Therefore, various scientific reports have been devoted to their potential industrial applications.

However, their use in the food industry as added-value ingredients is drastically limited, due to the characteristics of low solubility in an aqueous environment and due to their susceptibility to degradation [25,26]. Therefore, design strategies are needed to protect carotenoids from chemical and environmental factors prior to industrial applications [74,75].

Among the numerous methodologies used, encapsulation is one of the prominent approaches adopted in food processing industries [76,77].

**Figure 4:**
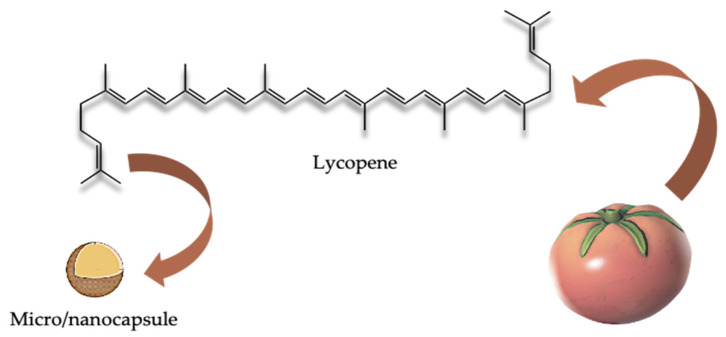
Microcapsules as delivery systems to improve the solubility and bioaccessibility of lycopene.

For example, Mihalcea et al. recently aimed to improve the solubility and bioaccessibility of lycopene, using the encapsulation technique to prepare microcapsules as delivery systems [78] (Figure 4).

Microencapsulation is a process that enables the design of a wall that encloses the biologically active compounds and protects them against external factors while offering a controlled release, masking the bitter taste, and preserving fragrance [79]. The authors in this study firstly performed an SC-CO_2_ extraction of lycopene from tomato peels. Then, the extracts with a higher lycopene content (93% and 76%) were used to produce microcapsules, using whey protein isolates and acacia gum as microencapsulating agents, through two different encapsulation techniques: complex coacervation and freeze-drying [80]. Complex coacervation is a process that involves the interaction of oppositely charged polyelectrolytes in aqueous form, which offers high encapsulation efficiency and a relatively low cost of processing [81]. Freeze-drying is the most commonly used method of encapsulation based on the dehydration by sublimation of a frozen sample, which is suitable for sensitive bioactive compounds because substances are not exposed to high temperature [82,83]. The prepared powders showed an antioxidant activity of about 9.37 ± 0.48 mmol TEAC/g dry weight (DW), and a good lycopene retention (48%) was found after 21 days at 25 °C, thus resulting in a pronounced improvement of lycopene stability.

The same research group also evaluated the preparation of lycopene microcapsules using an extract with 72% lycopene obtained via an ultrasound-assisted procedure from tomato peels [84]. The microencapsulation afforded a powder with an antioxidant activity of 2.15 ± 0.02 mmol Trolox/g DW and a retention of 63% in lycopene after storage at 4 °C in the dark for 14 days. Next, different ratios of the microcapsules were added as food ingredients into a dressing formulation based on sunflower oil and soy milk. The added value of the products was demonstrated through a higher antioxidant activity supporting the assumption that microencapsulation allows obtaining stable powders as functional ingredients. In a different study, [85] the encapsulation of extracted lycopene and β-carotene was realized with various materials such as soy protein, pea protein, inulin, and gum arabic using a freeze-drying method. Encapsulates using gum arabic showed the highest encapsulation efficiency of β-carotene (53.47%), while the encapsulate prepared with soy protein showed the highest encapsulation efficiency of lycopene (51.44%).

Although the microencapsulation techniques are efficient, it is necessary to decrease the size of the encapsulated material to the nano range (<0.10 μm) to overcome some drawbacks such as poor aqueous solubility and low bioavailability in the human body. In this context, nanoencapsulation technology has the potential to meet industry challenges concerning the effective delivery of health functional compounds [86,87]. This quite challenging technique was very recently proposed by Mishra and Kumari [88] by preparing lycopene nanoparticles. The nano-formulations were made using a nanoprecipitation method with ethanol as a solvent and poly(vinyl alcohol) (PVA) as a stabilizer; they were then assessed for their antidiabetic activity in experimental animals. It was observed that the oral administration of lycopene nanoparticles to diabetic rats showed a significant decrease (*p* < 0.001) in elevated blood sugar levels and significantly decreased the oxidative stress by changing oxidative stress biomarkers. The overall results suggest the possible use of nanoparticulate systems to decrease the treatment dosage.

## 4. Applications in the Food Industry

**Figure 5:**
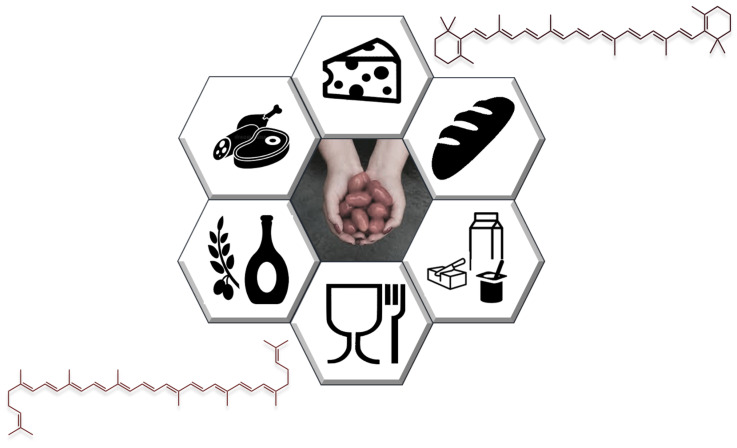
Applications of carotenoids derived from tomato in the food industry.

Among many other possible uses of bioactive compounds derived from tomato waste, their upcoming application in the food industry as food additives continues to be the most sought to fulfill consumer demand for natural and preserved healthy foods [89,90] (Figure 5).

In a recent study, the content of various nutrients and bioactive compounds in the waste coming from the tomato processing industries was determined with the aim of enabling the development of new alternatives for recycling this valuable byproduct [91]. The results confirmed that dried tomato wastes contain considerable amounts of lycopene (510.6 mg/kg) and β-carotene (95.6 mg/kg) and exhibit good antioxidant properties (6.8 mmol Trolox/kg) due to the additional presence of phenolic compounds, as already remarked by many researchers [92,93].

Tomato carotenoids can provide or enhance nutritional, sensory, and functional properties in food formulations either by direct or by indirect application, for example, in active food packaging [94].

In this sense, an interesting review by Domínguez et al. [95] reported an extensive list of studies which reformulated different meat products by including tomato extracts as natural additives in their composition. In fact, meat fat is highly susceptible to oxidation, especially the unsaturated fatty acid fraction. Various authors agree that the use of tomato byproduct extracts offers many advantages: improved nutritional quality, reduced lipid oxidation, and increased shelf-life of meat products, while retaining or increasing sensory properties and overall acceptability.

In addition to meat, milk products play a huge role in the traditional diet of people; however, following prolonged storage, these products undergo changes in odor and taste due to several biochemical deterioration reactions [96].

Zayan et al. evaluated the possible application of extracted lycopene oil of tomato peels waste as a convenient alternative to butter in processed cheese manufacturing [97]. In particular, they added different percentages of lycopene oil during cheese production and made a comparison with cheeses produced only with butter. The reformulated cheese showed increased lycopene content, antioxidant activity, meltability, and sensorial characteristics, suggesting that the substitution can be useful for decreasing saturated fats in dairy products.

Abid et al. evaluated the potential use of a lycopene-rich extract, from tomato processing byproducts, in extending the shelf-life of a traditional Tunisian butter [98]. The storage stability (during 60 days of storage at 4 °C) of the enriched butter was clearly increased due to the strong antioxidant activity that inhibited the formation of peroxide and conjugated dienes, as well as the breakdown of unsaturated fatty acids into oxidation products. In a similar study, the potential use of lycopene as a natural antioxidant added to Jordanian traditional sheep butter was demonstrated [99]. The overall results showed that natural lycopene may have a role in stabilizing the butter formulations, along with the benefit of adding a functional nutrient into such product.

Among other examples that might be described, important studies have been conducted in recent years on the enrichment of oils with carotenoids extracted from dry tomato waste. Noura et al. used various vegetable oils as alternative solvents in order to obtain oils enriched in carotenoids with improved thermal and oxidative stability. The extraction of dry tomato waste in vegetable oil resulted in colored functional oils with significant antioxidant activity when ingested as part of a dietary regime [100]. Accordingly, Freitas et al. evaluated the validity of carotenoids from industrial tomato wastes on the enrichment of soybean oil [101]. The so-treated soybean oil was subjected to heating in Rancimat (180 °C/5 h), revealing that tomato extract has a protective effect on industrialized oils, which retained 88% antioxidant compounds after the heating.

Natural lycopene was further applied as a functional ingredient to some bakery products [102]. For example, the addition of 3–5% lycopene extract to cake and cookies enhanced their antioxidant capacity, color, and sensory properties. Similarly, Mehta et al. [103] proposed the valorization of tomato processing byproduct by partial replacement of refined flour in bread and muffin. The supplemented bakery products showed a significantly improved shelf-life stability, as well as an increased antioxidant activity (48.7% and 45.3%) in comparison to the controls (11.2% and 9.3%). The high levels of phenolic compounds and lycopene in tomato skins compared to wheat flour could have been the reason for the increased antioxidant activity in bread and muffin. Thus, the incorporation of tomato waste-derived carotenoids is appealing for the possibility of improving the nutritional quality and chemical stability of numerous foodstuffs.

An attractive and sustainable valorization of food waste containing antioxidant compounds is its application for the development of food packaging materials [104]. Natural antioxidants can be valuable compounds to produce protective edible coatings in food products or films for food packaging purposes. A study carried out by Gallego et al. aimed to evaluate the effect of a gelatin coating enriched with antioxidant tomato byproduct hydrolysate on the quality of pork meat during cold storage [105]. Gelatin is an edible biopolymer largely employed as a coating due to its low cost, high availability, and functional properties, which can be used as a carrier of antioxidant compounds to retard oxidation processes and, thus, extend the shelf-life of highly perishable products [106]. Gelatin coatings showed a decrease in lipid oxidation of meats during storage, and enriched gelatin-coated meats after cooking presented high antioxidant activity. These results suggest a positive role of gelatin coating enriched with antioxidant tomato byproducts in extending the shelf-life of meat during storage.

Active packaging prepared with biopolymers or byproduct extracts represents a viable option for extending shelf-life and protecting food against degradation. However, they exhibit weak mechanical attributes and high water solubility and permeability compared to commercially available polymers. Therefore, Szabo et al. used tomato byproduct extracts for the development of new bioactive formulations for food packaging and evaluated their resistance and antibacterial activity [107].

In the study, films containing poly(vinyl alcohol) (3% *w*/*v*) and chitosan (1% *w*/*v*) were enriched with tomato byproduct extract, which not only significantly contributed to the resistance of the films but also showed good antibacterial activity toward *S. aureus* and *P. aeruginosa*, with an MIC of <0.078 mg DW/mL. Therefore, tomato processing wastes could represent a low-priced antimicrobial agent implied in food packaging and storage.

## 5. Applications in the Pharmaceutical and Cosmetic Industry

In addition to the widespread valorization by the food industry, there is an increasing opportunity to use tomato waste to obtain a functional extract with pharmacological properties.

In this regard, as tomato has been associated with a reduced risk of cardiovascular disease, several studies evaluated the possible use of tomato extracts for the development nutraceutical formulations. Concha-Meyer et al. evaluated the antithrombotic properties of extracts obtained from different tomato pomace compositions (whole, seedless, and seeds) [108]. The extracts were prepared via ultrasound-assisted treatment using water or ethanol/water (1:1) as the solvent. It was observed that the time of sonication and the solvent used in the extraction had a significant role in anti-platelet aggregation activity, showing that the sonication cycles only improved this activity in the seedless extracts due to the presence of greater amounts of flavonoids, well known for their potential therapeutic effect against CVD.

Recent clinical evidence of the bioactive properties of carotenoids has shown that these compounds may play a key role in the treatment of diabetes by improving insulin resistance, which has been indicated as a major risk factor for the development of type 2 diabetes mellitus. Novellino et al. [109] prepared a nutraceutical formulation including a dried tomato peel powder that was then tested on healthy human subjects in order to evaluate its effects on glycemic and insulinemic responses to a standard glucose drink. The nutraceutical formulation was shown to be able to influence postprandial glycemia through an insulin-saving mechanism, producing lower peak plasma concentrations with respect to the reference glucose solution.

Even the cosmetic industries in recent years are exploiting food-processing residues containing bioactive compounds to add value to their preparations. The inclusion of carotenoids is designed not only to increase the shelf-life of the cosmetic products but also to provide skin protection against oxidative damage [110]. Nevertheless, one of the main issues is that such an inclusion can lead to a change in the color and smell of the formulation, thus affecting acceptability. Accordingly, Costa et al. prepared microemulsions and macroemulsions for topical use with the incorporation of lycopene-enriched extracts from tomato waste [111]. After the evaluation of the presence of lycopene in the formulation, they carried out a sensory analysis to assess the odor and color perception and the acceptability of such systems. Overall results showed that lycopene extract conferred a yellowish color and an undefined odor to formulations, and the information about the formulation composition, i.e., the presence of an antioxidant compound, increased the acceptability of the cosmetic products.

## 6. Concluding Remarks

The growing attention to environmental problems requires food and pharmaceutical industries to adopt safe and sustainable technologies to be applied to the recovery, recycling, and valorization of byproducts, which pose serious problems in terms of disposal and potential pollution and represent a loss of precious biomass and nutrients.

The recovery of carotenoids and, above all, lycopene, considered one of most potent antioxidants, from tomato waste has gained a special interest in recent years, as it has been clearly shown that it may provide marked benefits for the food, pharmaceutical, and cosmetic industries. This review presented the up-to-date trends of research on the extraction and valorization of carotenoids from tomato processing waste. On a whole, the eminent literature reported highlighted how the tomato wastes are promising cheap sources of antioxidants to be recovered in order to implement a sustainable strategy that addresses the current challenges of the industrialized world.

## Figures and Tables

**Table 1:** Latest and innovative extraction methods.

Extraction Method	Extractive Principle	Extraction Sample	Treatment Conditions	Lycopene Extraction Yield	β-Carotene Extraction Yields	Total Carotenoids Extraction Yields	Ref.
Enzyme-assisted extraction	Enzyme-assisted hydrolysis of polysaccharide network of primary cell wall	Bulgarian tomato peel	Pretreatment with a mixture of cellulase (100 U·g^−1^) and endo-xylanase (400 U·g^−1^) for 4 h at 50 °C followed by the extraction with acetone for 30 min at 20 ± 1 °C (solid/liquid ratio: 1:30)	15.44 mg/100 g (d.w.)	35.85 mg/100 g (d.w.)	-	[43]
		Tomato waste (peel and seeds)	Pretreatment with a mixture of cellulolytic and pectinolytic enzymes for 5 h at 40 °C (enzyme: substrate ratio = 0.2 mL/g) followed by extraction with acetone: ethyl acetate mixture (solvent: substrate ratio = 5 mL/g) for 1 h at RT	11.5 mg/g	-	-	[44]
Supercritical Fluid Extraction (SFE)	Supercritical conditions enhance the solvating power of the solvent, thus favoring the extraction of intracellular compounds.	Tomato peels	Extraction at 50–80 ° C, pressures of 300–500 bar and flow rates of 4−6 g CO_2_/min for 105 min	1198 ± 71.86 mg/kg (d.w.)	27.94 ± 0.06 mg/kg (d.w.)	-	[49]
		Tomato pomace	57 °C, 40 MPa, and 1.8 h of extraction	28.64 mg/100 g d.w.	-	-	[50]
		Tomato flesh and peels	59 °C, 350 bar, 15 g/min CO_2_, 15.5% (*v*/*v*) ethanol as co-solvent, for 30 min	98.5 ± 2.1 in Tomato flesh and 92.5 ± 2.2 tomato peels (%, *w*/*w*) (d.b)	99.0 ± 2.8 in tomato flesh 96.9 ± 1.7 in tomato peels (%, *w*/*w*) (d.b)	-	[51]
Pulsed Electric Field (PEF)	Lethal damage to cells or induce sub-lethal stress by transient permeabilization of cell membranes and electrophoretic movement of charged species between cellular compartments.	Tomato peels	PEF (0.25–0.75 kV/cm, 1 kJ/kg) and SB (1 min at 60 °C) pretreatment followed by extraction in acetone (4 h at 25 °C).	-	-	37.9 mg/100 g (f.w.)	[53]
		Industrial tomato peels residues	PEF pretreatment (5 kV/cm, 5 kJ/kg) before solvent extraction process with either acetone or ethyl lactate (1:40 g/mL) at 25 °C extraction time set at 240	11820 ± 141 mg/kg (d.w.) in acetone and 6311 ± 254 mg/kg (d.w) in ethyl lactate	-	-	[56]
Ultrasound-assisted Extraction (UAE)	Disruption of cells by shock waves from cavitation bubbles, thus facilitating mass transfer hence an absolute increase in the extraction yield and kinetics.	Tomato pomace freeze-dried	Ultrasound-assisted extraction with Ethyl Lactate/Ethyl Acetate (7:3) solvent mixture, 100 mL/g solvent: sample ratio for 20 min	1334.8 µg/g (d.w.)		-	[62]
		Laboratory and industry generated tomato waste by-products	Combined treatment of freeze drying and ultrasonication (45 min at 50 Hz) at RT	138.82 ± 6.64 µg/g (f.w.)	-	-	[63]
		Tomato pomace	Menthol: lactic acid (8:1) DES, 120 mL/g solvent: sample, and 10 min at 70 °C in an ultrasound water bath with fixed frequency (40 kHz) and power (100 W)	1446.6 µg/g (d.w.)	-	-	[55]
Hydrocolloidal system	Water-induced complexation of lycopene in the presence of pectin, a bioactive compound also presents in tomato pomace	Tomato pomace	water–induced complexation of carotenoid and pectin by 851 rpm of stirring speed for 10 min and with 4.69% of processed wet samples	-	-	9.43 mg/100 g (wet) 4332.32 mg/100 g [carotenoid–pectin complex]	[68]
Microwave-assisted extraction (MAE)	Heating causes the evaporation of the mixture, causing moisture to, which generates tremendous pressure and the rupture of cells. This facilitates the release of the desired intracellular contents	Tomato waste	300 w power microwave, 60 s, 95% ethanol, with a temperature not exceeding 77 °C	5.74 mg/100 g (d.w.)	4.83 mg/100 g (d.w.)	-	[70]
Naviglio extractor	Forced extraction of the not chemically bound compounds contained in the solid matrix in a suitable solvent, by the generation of a gradient pressure between the inner and the outlet of solid matrix	Tomato skin	60 extraction cycles from tomato waste in deionized water using a Naviglio Extractor. Each cycle consisted of a 2-min static phase, followed by a dynamic phase and a delay time of 12 s before a new cycle started. 4 h is the time required for the extraction	5 mg/200 g (d.w.)	-	-	[71]
